# Synergistic effects of epigallocatechin gallate and l-theanine in nerve repair and regeneration by anti-amyloid damage, promoting metabolism, and nourishing nerve cells

**DOI:** 10.3389/fnut.2022.951415

**Published:** 2022-08-10

**Authors:** Xinya Xie, Juan Wan, Xin Zheng, Wenjing Pan, Jiayi Yuan, Baozhu Hu, Meiyan Feng, Zhonghua Liu, Shuxian Cai

**Affiliations:** ^1^National Research Center of Engineering Technology for Utilization of Botanical Functional Ingredients, Hunan Agricultural University, Changsha, China; ^2^Key Laboratory of Ministry of Education for Tea Science, Hunan Agricultural University, Changsha, China; ^3^Co-Innovation Center of Education Ministry for Utilization of Botanical Functional Ingredients, Hunan Agricultural University, Changsha, China

**Keywords:** EGCG, L-theanine, synergistic effects, β-amyloid stress, neuroinflammation, cell cycle regulation

## Abstract

Green tea has significant protective activity on nerve cells, but the mechanism of action is unclear. Epigallocatechin gallate (EGCG) and *N*-ethyl-L-glutamine (L-theanine) are the representative functional components of green tea (*Camellia sinensis*). In this study, an AD model of Aβ_25–35_-induced differentiated neural cell line PC12 cells was established to study the synergistic effect of EGCG and L-theanine in protecting neural cells. The results showed that under Aβ_25–35_ stress conditions, mitochondria and axons degenerated, and the expression of cyclins was up-regulated, showing the gene and protein characteristics of cellular hyperfunction. EGCG + L-theanine inhibited inflammation and aggregate formation pathways, significantly increased the percentage of G0/G1 in the cell cycle, downregulated the expression of proteins such as p-mTOR, Cyclin D1, and Cyclin B1, upregulated the expression of GAP43, Klotho, p-AMPK, and other proteins, promoted mitochondrial activity and energy metabolism, and had repair and regeneration effects on differentiated nerve cells. The synergistic mechanism study showed that under the premise that EGCG inhibits amyloid stress and inflammation and promotes metabolism, L-theanine could play a nourish nerve effect. EGCG + L-theanine keeps differentiated nerve cells in a quiescent state, which is beneficial to the repair and regeneration of nerve cells. In addition, EGCG + L-theanine maintains the high-fidelity structure of cellular proteins. This study revealed for the first time that the synergistic effect of EGCG with L-theanine may be an effective way to promote nerve cell repair and regeneration and slow down the progression of AD. Our findings provide a new scientific basis for the relationship between tea drinking and brain protection.

## Introduction

Proteotoxic stress, mitochondrial dysfunction, and genomic instability lead to cellular hyperactivity and are closely associated with age-related degenerative diseases such as atherosclerosis, type 2 diabetes, osteoporosis, and Alzheimer’s disease (1–3). Under various stressful conditions, cells promote mTOR or other growth factors sensitive growth-stimulating pathways, causing cells to exhibit hyperactivity.

Following damage to the genome and epigenome of cells, many senescence-related secretions are mainly transcribed through NF-κB, secreting interleukins, growth factors, proteases, cytokines, matrix-degrading enzymes, and metalloproteinases into the extracellular space. Furthermore, it mediates insulin resistance, blocks cellular signaling feedback, accelerates senescence in neighboring cells, and mediates the development of senescent phenotypes. Pathological amyloid Aβ is the most neurotoxic agrin with a high aggregation rate. Many drugs can effectively inhibit the formation of Aβ, but clinical studies have no effect. Earlier studies identified Aβ amyloid plaques in the extracellular matrix, and recent studies have also found low levels of amyloid plaques in the intracellular Golgi apparatus, endoplasmic reticulum, and mitochondria (4, 5). Nerve cell axons are rich in mitochondria and prone to degeneration of nerve cells and axons under β-amyloid stress (6).

Alzheimer’s disease is a neurodegenerative disease characterized by dysregulation of the neuronal cell cycle, leading to cell death (7). Cell cycle markers are present in degenerating AD neurons in G1, S, G2, or M phases, indicating that terminally differentiated neurons re-enter the cell cycle. When the cell cycle is reactivated, the function and integrity of terminally differentiated neurons are disrupted. Furthermore, inappropriate activation of cyclins alters the function of DNA-binding proteins, thereby affecting the overall structure and further DNA damage by exacerbating oxidative damage by various endonucleases (8). Therefore, AD and other related neurodegenerative diseases can be effectively treated by inhibiting amyloid stress, regulating cell cycle, keeping cells in a quiescence state, improving energy metabolism, and promoting axonal growth (9, 10).

**FIGURE 1:**
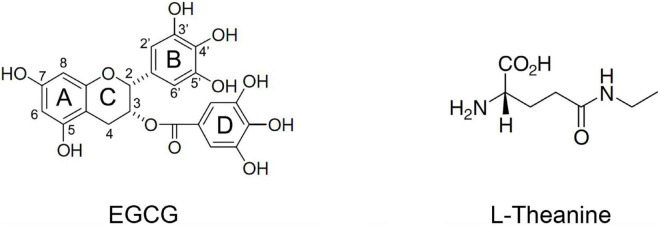
Molecular structures of the active ingredients Epigallocatechin gallate (EGCG) and *N*-ethyl-L-glutamine (L-theanine) in tea.

Currently, there is much literature on the neuroprotective effects of green tea (*Camellia sinensis*) and its core functional components Epigallocatechin gallate (EGCG) and *N*-ethyl-L-glutamine (L-theanine) (11–14). The molecular structures of EGCG and L-theanine are shown in Figure 1. EGCG is a flavonoid that belongs to the gallate-esterified flavan-3-ols. EGCG accounts for about 9–13% of the dry tea weight of tea leaves, and 50–80% of the total catechins. EC, EGC, and ECG each account for about 5–15% of the total catechins (15). EGCG significantly protects biological macromolecular structures such as proteins and phospholipids by inhibiting the formation of β-sheet, a toxic seed structure of aggregates (16, 17). EGCG is a mitochondria-targeted drug that modulates mitochondrial metabolism, including mitochondrial biogenesis, and mitochondrial-mediated cell cycle and apoptosis (18, 19). In addition, EGCG can also promote axonal growth in PC12 cells (20). L-theanine, a non-protein water-soluble amino acid, is characteristically found in tea plants (21). L-theanine content accounts for about 1–2% of the dry weight of tea leaves, and the content of L-theanine in high-theanine tea tree varieties is about 4% or more (22). There are 26 amino acids identified in tea, of which 6 are non-protein amino acids. L-theanine accounts for about 50–60% of the total amino acids in tea (22). L-theanine has cerebral protective activities such as anti-anxiety, anti-depression, memory promotion, prevention of vascular dementia, and nutritional nerve (23–26). L-theanine can inhibit glutamate receptors, increase intracellular glutamine and glutathione concentrations, attenuate oxidative damage, and have neuroprotective effects (24). In the SAPM8 brain aging model, L-theanine significantly inhibits the concentration of Aβ42 and down-regulates the Aβ42 formation pathways such as PS1, BACE1, and p16 (27).

Epigallocatechin gallate is mainly stored in the vacuoles of fresh tea leaves and has protective effects on tea trees such as anti-ultraviolet rays and anti-insect pests. L-theanine is synthesized in the roots of tea trees, and has a high content in the actively growing tea buds and other tissues, providing carbon and nitrogen sources for the growth of tea trees (28, 29). The repairing effect of EGCG and the growth-promoting effect of L-theanine are beneficial to the growth of tea tree shoots. This study speculates that EGCG and L-theanine have a synergistic effect on neuroprotection. PC12, a cell line derived from rat pheochromocytoma, has been widely used as an *in vitro* model to study neuronal apoptosis and differentiation (30). We established an AD cell model through PC12 cells to study the synergistic protective effect of EGCG and L-theanine on differentiated neural lineage cells.

## Materials and methods

### Materials

Both EGCG (≥99%) and L-theanine (≥99%) were purchased from Sigma-Aldrich (St Louis, MO, United States). In addition to the secondary antibody (Abcam, CA, United Kingdom), the following primary antibodies were used for western blot analysis: anti-GAPDH, anti-Histone H3, anti-CyclinD1, anti-CyclinB1, anti-IL6, anti-AMPK, anti-mTOR, anti-Sirt1, anti-NF-κB, anti-Nrf2, and anti-KEAP1 (Cell Signaling Technology, Boston, United States), anti-Klotho (Novusbio, Colorad, United States), both RAGE and β-Amyloid (Santa Cruz Biotechnology, San Cruise, United States), anti-p62 (Epitomics, Burlingame, United States), anti-4-HNE (Millipore, Boston, United States), Anti-Multi Ubiquitin mAb (MBL, Tokyo, Japan). Western chemiluminescent horseradish peroxidase substrate (Thermo Fisher Scientific, Waltham, MA, United States), protease inhibitor mixture (Beyotime, Shanghai, China), BCA protein assay reagent (Thermo Fisher Scientific, Waltham, MA, United States), and radioimmunoprecipitation assay (RIPA) lysis buffer (KeyGEN BioTECH, Nanjing, China) were also used in the experiments.

### Incubation and preparation of different Aβ_25–35_ protein samples

Epigallocatechin gallate (1 mM), L-theanine (1 mM), and EGCG (1 mM) + L-theanine (1 mM) were mixed with Aβ_25–35_ (1 mM) at a concentration of 1 mM, respectively, and incubated in a 37°C incubator for 7 days (31). After incubation, they were diluted 20 times and added to cells for use. That is, the final concentrations used in cell experiments were 50 μM Aβ_25–35_, 50 μM Aβ_25–35_/50 μM EGCG, 50 μM Aβ_25–35_/50 μM L-theanine, and 50 μM Aβ_25–35_/50 μM EGCG + 50 μM L-theanine. In the description of the results that follow in the article, 50 μM Aβ_25–35_, 50 μM Aβ_25–35_/50 μM EGCG, 50 μM Aβ_25–35_/50 μM L-theanine, and 50 μM Aβ_25–35_/50 μM EGCG + 50 μM L-theanine are abbreviated as Aβ_25–35_, Aβ_25–35_/EGCG, Aβ_25–35_/L-theanine, and Aβ_25–35_/EGCG + L-theanine, respectively.

### Fluorescence detection of thioflavin T

Thioflavin T fluorescent probes bind to β-sheet-rich molecules to emit fluorescence. ThT fluorescent probes are ideal for detecting amyloid aggregates in protein misfolding research (32, 33). Mixed 10 μL of aliquots of Aβ_25–35_ solution (1 mM) with 90 μL of ThT (80 μg/mL) in PBS. The fluorescence was measured at λex and λem of 440 and 485 nm, respectively, using a fluorescence microplate reader (Thermo, Waltham, MA, United States).

### Cell culture

Rat pheochromocytoma cell lines (PC12 cells) were from Peking Union Medical College, Cell Bank (Beijing, China) and grown in Dulbecco’s modified Eagle medium (DMEM) (Cromwell, CT, United States) containing 10% fetal bovine serum (FBS) (BI, IL), 100 U/ml penicillin, and 100 μg/ml streptomycin at 37°C in a humidified incubator with 95% air and 5% CO_2_. During cell culture, cells were passaged approximately every 2 days.

### Aβ_25–35_ insult, drug treatment, and 3-(4,5-dimethyl-2-thiazolyl)-2,5-diphenyl tetrazolium bromide assay

PC12 cells were seeded in a 96-well cell culture plate at a density of 10^4^/well and cultured for 24 h. Then the cells were treated with different Aβ_25–35_ protein samples (Aβ_25–35_, Aβ_25–35_/EGCG, Aβ_25–35_/L-theanine, and Aβ_25–35_/EGCG + L-theanine) for 24 h. Cell viability was detected by MTT colorimetric assay. Briefly, the supernatants were exchanged with a medium containing 0.5 g/ml MTT for 4 h, the superments were removed, and 150 μL/well dimethyl sulfoxide (DMSO) was added. The absorbance was detected at 570 nm. The relative cell viability was expressed as the mean percentage of absorbance in treated vs. control cells. The value of the control was set at 100%.

### Transmission electron microscopic examination

Cell samples from different treatment groups were collected and double-fixed in 2.5% glutaraldehyde solution containing Millonig’s phosphate buffer (pH = 7.3). Examined and photographed on a Hitachi HT-7700 electron microscope after double staining with 3% uranyl acetate and lead nitrate.

### Flow measurement of cell cycle and apoptosis

After collecting cells, washed twice with pre-chilled PBS. The cell cycle determination steps were as follows: added 2 mL of 70% ethanol, vortex to mix the cells, incubated at −20°C for 2 h, removed the ethanol by centrifugation, and washed twice with PBS, divided the cells into 5 mL flow cytometry tubes, added 500 μL PI/Rnase staining buffer, mixed the cells, incubated at room temperature in the dark for 15 min, stored at 4°C, and detected by flow cytometry within 1 h. The steps for apoptosis assay were as follows: added 1 × cell binding buffer, divided the cells into 5 mL flow cytometry tubes, each tube contained 1 × 10^5^ cells, added 5 μL each of FITC Annexin V and PI dye, and protected from light until incubated for 15 min at room temperature, added 400 μL 1 × cell-binding buffer to each tube, and detected by flow cytometry (FACSCalibur system, BD Biosciences) within 1 h.

### Fourier transform infrared spectroscopy spectral analysis

Cell samples from different treatment groups were collected for freeze-drying and infrared spectroscopy analysis. Cells protein samples were prepared by freeze-drying method, and vacuum freeze-dried at −42°C for 24 h. Samples were detected by Fourier transform Infrared spectroscopy (FTIR) (34, 35). The spectral range was 4000–400 cm^–1^. It was recorded in reflectance mode with a spectral resolution of 4 cm^–1^, accumulating 64 scans per spectrum. Amide I bands (1700–1600 cm^–1^) were used to determine the secondary structure of proteins in cell samples from different treatment groups, and spectra of original untreated spectra were synthesized and analyzed using omnic software (Thermo Nicolet, Waltham, United States).

### JC1 detection of cell mitochondrial membrane potential

Mitochondrial membrane potential was detected using a fluorescent dye mitochondrial membrane potential detection kit (JC-1) staining kit (Beyotime, Shanghai, China) (36). PC12 cells were seeded on 24-well cell slides at a density of 5 × 10^4^/well, and the cells were cultured according to the experimental method in Sections “Cell culture” and “Aβ_25–35_ insult, drug treatment, and 3-(4,5-dimethyl-2-thiazolyl)-2,5-diphenyl tetrazolium bromide assay.” Subsequent experimental operations were carried out in strict accordance with the kit instructions. After the experiment, the staining of cells in different treatment groups was observed by s fluorescence microscope (Zeiss, Axio scope. A1). In the dual-color staining of JC-1, the higher the ratio of red/green fluorescence intensity, the higher the mitochondrial membrane potential.

### DAPI fluorescent staining

After cell experiment, the cells were washed three times in the dark with PBS, mounted with Antifade Mounting Medium with DAPI (Beyotime, Shanghai, China), and then photographed with a fluorescence microscope (Zeiss, Axio scope. A1).

### LDH cytotoxicity assay

The lactate dehydrogenase kit (Beyotime, Shanghai, China) detected lactate dehydrogenase activity released by cells in different treatment groups. PC12 cells were seeded in a 96-well cell culture plate at a density of 1 × 10^4^/well, and cells were cultured according to the experimental methods in Sections “Cell culture” and “Aβ_25–35_ insult, drug treatment, and 3-(4,5-dimethyl-2-thiazolyl)-2,5-diphenyl tetrazolium bromide assay.” After the experiment, the cell culture medium of different treatment groups was collected and centrifuged (400 g, 5 min) to remove dead cells in the culture medium. Finally, the LDH in the culture medium was detected strictly according to the operation method of the kit.

### Adenosine triphosphate detection

Usually, a drop in ATP levels indicates impaired or decreased mitochondrial function (37). PC12 cells were seeded in a 6-well culture plate at 1.5 × 10^5^/well, and cultured according to the experimental methods in Sections “Cell culture” and “Aβ_25–35_ insult, drug treatment, and 3-(4,5-dimethyl-2-thiazolyl)-2,5-diphenyl tetrazolium bromide assay.” After the experiment, the medium was discarded, washed twice with pre-cooled PBS, 200 μL of cell lysate was added to each well, centrifuged (4°C, 12000 rpm, 20 min), and the supernatant was collected. The subsequent ATP detection experiments were performed according to the Adenosine triphosphate (ATP) detection kit (Beyotime, Shanghai, China).

### Western blotting assay

After cell experiments were completed, cells were washed twice with pre-chilled PBS, an appropriate lysis buffer containing 1 × cocktail (Fermentas) and RIPA buffer (Pplygen), and the cells were lysed for 30 min on ice, vortexing every 10 min. The supernatant was collected by centrifugation (4°C, 12000 rpm, 20 min). Protein concentrations were determined using the BCA kit (Thermo, Waltham, United States), and equal amounts of protein (30 μg) per sample were separated by SDS-PAGE electrophoresis. After electrophoresis, the proteins on the gel were transferred to PVDF (Millipore, Boston, United States) membranes and blocked with TBST buffer containing 5% non-fat milk at room temperature for 1 h with slow shaking. Primary antibodies were added, overnight at 4°C. After washing with TBST for 5 min × 5 times at room temperature, the PVDF membranes were incubated with appropriate secondary antibodies for 1 h. After washing with TBST for 5 min × 5 times, the PVDF membranes were reacted with an enhanced chemiluminescence substrate (Piece Rockford, United States) for 2∼5 min. The luminescence signals were then detected by a chemiluminescence gel imaging system, and finally the optical density (OD) value of each band was analyzed using Image-J software.

### Enzyme-linked immunosorbent assay

The TNF-α expression levels in the cell protein samples of different treatment groups were detected strictly according to the ELISA kit (Jiangsu Feiya Biological Technology Co. Ltd.) operation method.

### BODIPY fluorescent staining

PC12 cells were seeded on 24-well cell slides at a density of 5 × 10^4^/well, and the cells were cultured according to the experimental method in Sections “Cell culture” and “Aβ_25–35_ insult, drug treatment, and 3-(4,5-dimethyl-2-thiazolyl)-2,5-diphenyl tetrazolium bromide assay.” After the experiment, the cells were washed twice with PBS, and 200 M l/well of fixative was added for 10 min. After washing twice with PBS, BODIPY staining solution (Invitrogen, New York, United States) was added and incubated for 30 min in an incubator. Finally, the staining of cells in different treatment groups was observed by s fluorescence microscope (Zeiss, Axio scope. A1).

### RNA sequencing and gene expression analysis

After the cell experiment, the collected cell samples were immediately pre-frozen in liquid nitrogen for 2 min, frozen in dry ice and sent to BGI (Shenzhen, China^1^) for transcriptome sequencing (RNA-seq) detection. The expression level of each gene was calculated using the number of fragments per kilobase of transcript (FPKM) after normalizing the expression level of each gene. Differentially expressed genes (DEGs) were screened by the screening conditions of | fold changes| ≥ 2 and *Q* value ≤ 0.05, and correlation analysis was performed in different treatment groups. In order to more comprehensively explore the mechanism of action from the transcriptome level, with |fold changes| ≥ 1.2 and *Q* value ≤ 0.05 as the screening conditions, more DEGs were screened for network interaction analysis. In addition, to explore the synergistic mechanism of EGCG and L-theanine, according to the DEGs of the Aβ_25–35_/EGCG + L-theanine treatment group, a heat map analysis of the expression of related genes in different treatment groups was performed. Finally, STRING^2^ and Cytoscape (United States, v3.8.2) software were used to analyze the network interaction and visualize visualization of DEGs in different blocks in the heat map.

### Statistical analysis

Using GraphPad Prism 8.01 software (GraphPad Software Inc., San Diego, United States), combined with Turkey’s multiple comparison test, a one-way ANOVA test was used to analyze the significance of differences. The results were expressed as mean ± standard deviation. The variation was judged significant at *p* < 0.05 and highly significant at *p* < 0.01.

## Results

### Epigallocatechin gallate and L-theanine synergistically protect PC12 cells from Aβ _25–35_-induced injury

**FIGURE 2:**
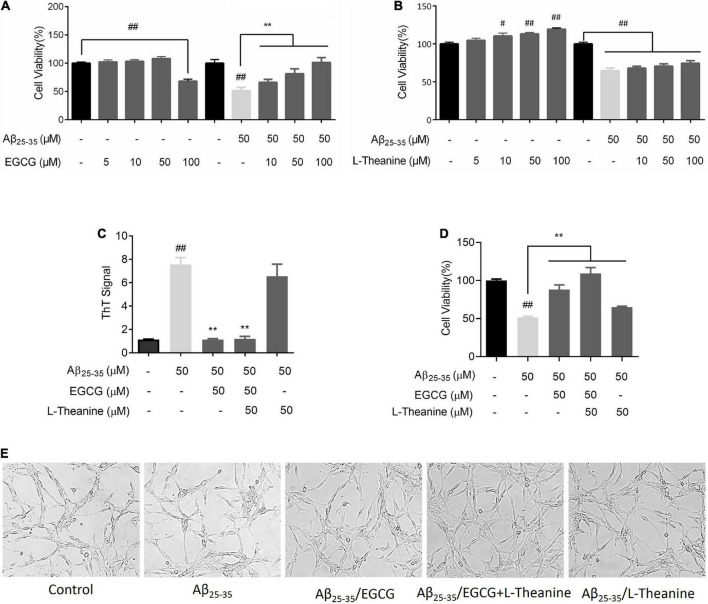
EGCG and L-theanine synergistically protect PC12 cells from Abeta_25–35_-induced injury. **(A)** MTT assay of cell viability in EGCG or Aβ_25–35_/EGCG treated group. **(B)** MTT detection of cells viability in L-theanine or Aβ_25–35_/L-theanine treated group. **(C)** ThT assay of different Aβ_25–35_ proteins samples. **(D)** MTT assay of cell viability in the synergistic group of EGCG and L-theanine. **(E)** White light photography of cell morphology. #*p* < 0.05, ##*p* < 0.01, compared with control group; **p < 0.01, compared with Aβ_25–35_ group, *n* = 6.

PC12 cells were incubated with different concentrations of EGCG or L-theanine for 24 h. EGCG had no significant effect on cell viability in the 50 μM concentration range, but significantly decreased cell viability at 100 μM concentration (*p* < 0.01). L-theanine promoted cell growth in a concentration-dependent manner in the 100 μM range. The above results revealed that EGCG had no growth-promoting effect on cells, and had cytotoxicity at a high concentration of 100 μM; while L-theanine had a cell growth-promoting effect at a concentration of 100 μM (Figures 2A,B).

Soluble oligopeptide β-sheet (A11+) structure, the seed structure of protein misfolding and protein aggregate formation, is the toxic core structure of amyloid (38, 39). Aβ_25–35_ is the neurotoxic fragment of Aβ_1–40_ and Aβ_1–42_ (40, 41). ThT fluorescence probe detection was performed on different Aβ_25–35_ (50 μM) protein samples. The results showed that the β-sheet (All+) structure of the Aβ_25–35_ group was significantly increased (*p* < 0.01); EGCG and EGCG + L-theanine had significant inhibitory activity on β-sheet (All+) structure formation (*p* < 0.01), while the inhibitory activity of L-theanine was not significant (Figure 2C). EGCG interacts with Aβ, apparently forming small unstructured Aβ aggregates (42). When Aβ_25–35_ was at 50 μM, EGCG had the best cytoprotective activity at 50 μM concentration. L-theanine from 10 to 100 μM had a significant effect on promoting cell growth. While under Aβ_25–35_ stress conditions, different concentrations of L-theanine had no cytoprotective activity, and the difference in activity was not obvious. Therefore, we determined the concentration of EGCG and L-theanine to be 50 μM each for subsequent synergy studies. Subsequent content abbreviated 50 μM Aβ_25–35_, 50 μM Aβ_25–35_/50 μM EGCG, 50 μM Aβ_25–35_/50 μM L-theanine and 50 μM Aβ_25–35_/50 μM EGCG + 50 μM L-theanine to Aβ_25–35_, Aβ_25–35_/EGCG, Aβ_25–35_/L-theanine and Aβ_25–35_/EGCG + L-theanine, respectively.

Different Aβ_25–35_ protein samples were incubated with PC12 cells for 24 h. The results of MTT and brightfield photography showed that Aβ_25–35_/EGCG, Aβ_25–35_/EGCG + L-theanine, and Aβ_25–35_/L-theanine significantly promoted cell activity, and the Aβ_25–35_/EGCG + L-theanine group had the strongest cell viability, and its activity was better than that of the control group (Figures 2D,E).

### Epigallocatechin gallate and L-theanine synergistically inhibit amyloidogenic and inflammatory pathways

**FIGURE 3:**
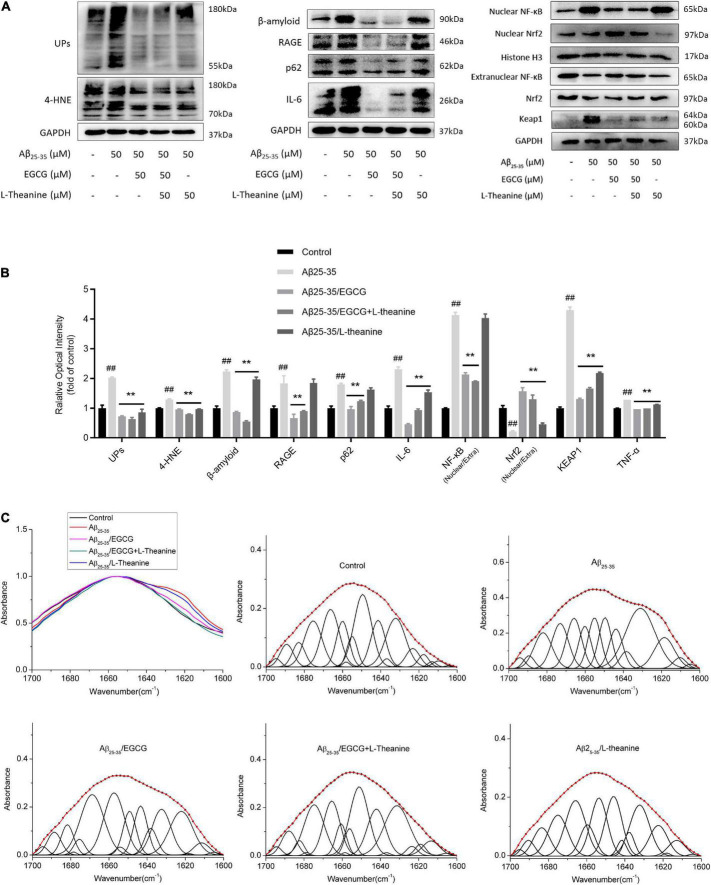
EGCG and L-theanine synergistically inhibit amyloidogenic and inflammatory pathways. **(A,B)** ELISA and Western-blotting detection and analysis of protein expression levels associated with aggregates formation and inflammation. Among them, ELISA detected TNF-α, and Western-blotting detected the remaining proteins. “Nuclear/Extra” indicates the degree of nuclear translocation of the associated proteins. **(C)** Amide I band (1700–1600 cm^–1^) infrared spectra and Gaussian fitting analysis of cell samples in different treatment groups. In the fitted plots, amide I maxima between 1675 and 1695 cm^–1^ are generally assigned to antiparallel β-sheet/aggregated strands; and amide I maxima of 1660–1670 cm^–1^, 1648–1660 cm^–1^, 1640–1648 cm^–1^, 1625–1640 cm^–1^, and 1610–1628 cm^–1^ are assigned to 3^10^-helix, α-helix, unordered strands, β-sheet, and aggregated strands, respectively (49). ##*p* < 0.01, compared with control group; **p < 0.01, compared with Aβ_25–35_ group, *n* = 3.

The most striking features of neurodegenerative diseases are the accumulation of protein aggregates and autophagic dysfunction (43, 44). Ubiquitin-conjugated Proteins (Ups) and p62-modified proteins can be diagnostic tools for the aggregate formation and autophagy dysfunction (45, 46). Western blotting analysis showed that Aβ_25–35_ group cells produced a large amount of β-amyloid, accumulated 4-HNE, Ups and p62-modified aggregates, up-regulated TNF-α content (*p* < 0.01) and RAGE protein expression, and increased NF-κB nuclear transcription level by about 75% (*p* < 0.01). The results showed that the Aβ_25–35_ group had increased cell aggregates formation and up-regulated inflammatory pathways (*p* < 0.01), while EGCG and EGCG + L-theanine had significant inhibitory effects, followed by L-theanine. Nrf2/KEAP1 is an endogenous antioxidant signaling pathway in cells, and Nrf2/KEAP1 is unstable and released under stress conditions (47). The level of nuclear translocation of Nrf2 protein decreased in Aβ_25–35_ group cells (*p* < 0.01), and the unbound keap1 protein increased (*p* < 0.01); EGCG, EGCG + L-theanine, and L-theanine promoted Nrf2 nuclear translocation (*p* < 0.01) and decreased the amount of unbound keap1 protein (*p* < 0.01) (Figures 3A,B).

β-sheet formation is a crucial early step in amyloidogenesis (48). The infrared absorption peak spectrum and fitting analysis results of the protein amide I band (1700–1600 cm^–1^) showed that (Figure 3C), the infrared absorption peaks of Aβ_25–35_ group shifted to low wavenumbers, and the β-sheet structure increased significantly, α-helix and disordered structures were reduced. EGCG significantly inhibited the formation of β-sheet structure and promoted the formation of α-helix structure, but still had a great influence on cellular protein structures; L-theanine inhibited the shift of the absorption peak to the lower band; the absorption peaks and secondary structures fitting peaks of the Aβ_25–35_/EGCG + L-theanine group were almost identical to those of the control group.

### EGCG + L-theanine targets cell cycle and promotes axonal growth

**FIGURE 4:**
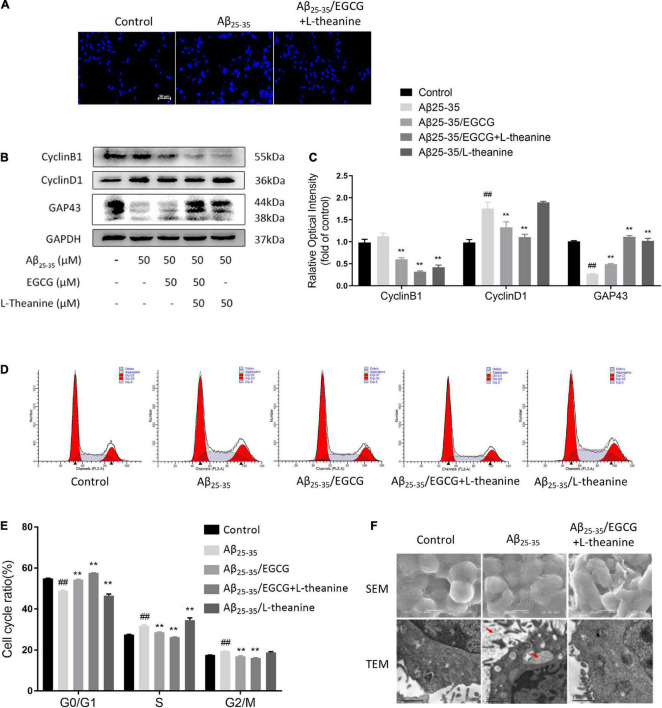
EGCG + L-theanine regulates cell cycle and promotes axonal growth. **(A)** DAPI nuclear staining observation (bar = 50 μm). **(B,C)** Western-blotting detection of Cyclin B1, Cyclin D1, and GAP43. **(D)** Flow cytometry analysis of cell cycle and apoptosis. **(E)** Statistical analysis of cell cycle. **(F)** Scanning electron microscopy (SEM) and transmission electron microscopy (TEM) analysis (red arrows point to the cellular fibrous aggregate fraction). ##*p* < 0.01, compared with control group; **p < 0.01, compared with Aβ_25–35_ group, *n* = 3.

The results of DAPI nuclear staining showed that the nuclei of the Aβ_25–35_ group had different morphologies, some nuclei were enlarged, and the staining was high, indicating severe DNA damage; the nuclei of Aβ_25–35_/EGCG + L-theanine group were uniform in morphology and light staining, same as the control group; the Aβ_25–35_/EGCG and Aβ_25–35_/L-theanine groups also had some improvement (Figure 4A).

Cell cycle control is closely related to neuronal axonal growth (50). Cyclin D1 and Cyclin B1 are markers of the G1 and G2 phases of the cell cycle, respectively (51). Cyclin D1 and Cyclin B1 are in a low expression state in differentiated neural cells to maintain cell differentiation state and normal function (52). The results of Western blotting showed that the protein expressions of Cyclin D1 and Cyclin B1 in the Aβ_25–35_ group were up-regulated, and the expression of Cyclin D1 increased by about 50% (*p* < 0.01); in Aβ_25–35_/EGCG and Aβ_25–35_/EGCG + L-theanine groups, Cyclin D1 and Cyclin B1 protein expressions were down-regulated (*p* < 0.01), and the Aβ_25–35_/EGCG + L-theanine group was the most significant; Cyclin B1 was down-regulated in the Aβ_25–35_/L-theanine group (*p* < 0.01) (Figures 4B,C).

The results of cell cycle detection showed that the percentage of S phase increased by about 5% in the Aβ_25–35_ group (*p* < 0.01). The Aβ_25–35_/EGCG and Aβ_25–35_/EGCG + L-theanine groups increased the percentage of G0/G1 phase (*p* < 0.01) and down-regulated the percentage of S phase (*p* < 0.01), which was more significant in the Aβ_25–35_/EGCG + L-theanine group. The S phase percentage of Aβ_25–35_/L-theanine was the highest (Figures 4D,E). The above results indicate that, under the condition of Aβ_25–35_ toxic stress, EGCG + L-theanine significantly promotes the formation of the quiescent state of cells, and is beneficial to the repair of differentiated nerve cells and the growth of axons.

The neurite growth factor GAP43 is a nerve-specific protein involved in nerve cell growth, synapse development and nerve cell regeneration (53). The expression of GAP43 protein in the Aβ_25–35_ group was decreased (*p* < 0.01); compared with the Aβ_25–35_ group, the expressions of the three tea component treatment groups were all up-regulated (*p* < 0.01), among which the Aβ_25–35_/EGCG + L-theanine group expression increased about 4-fold (Figures 4B,C).

The scanning electron microscopy (SEM) results showed that cells in the Aβ_25–35_ group had flattened cell bodies and atrophied axons; in the Aβ_25–35_/EGCG + L-theanine group, the cells were plump, with rich surface structures and thick axons. Transmission electron microscopy (TEM) results showed that in the Aβ_25–35_ group cells, there were fibrous aggregates in the cell membrane and intracellular organelles (the part indicated by the red arrow), mitochondrial vacuolation and nucleus heterochromatin aggregates were formed. In contrast, no fibrous aggregates were detected in the Aβ_25–35_/EGCG + L-theanine-treated group cells, and the cell status was significantly improved (Figure 4F).

### EGCG + L-theanine protects mitochondria and promotes energy metabolism

**FIGURE 5:**
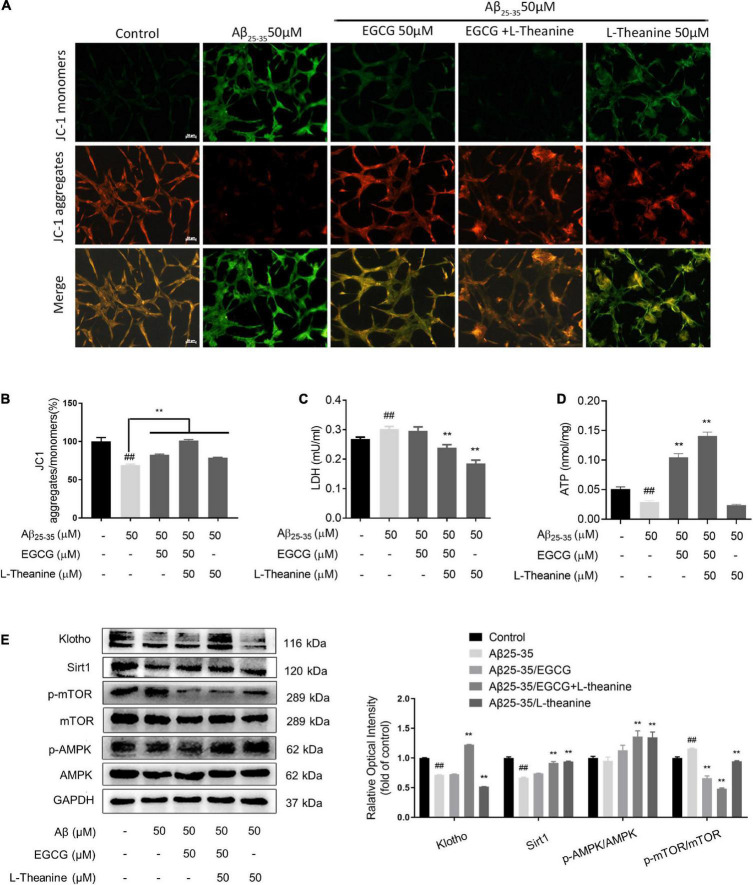
EGCG and L-theanine synergistically protect mitochondria and promote energy metabolism. **(A)** JC1 fluorescence staining of mitochondrial membrane potential. **(B)** JC1 staining statistics (bar = 50 μm). **(C,D)** Kit detection of LDH and ATP. **(E)** The expression levels of proteins related to mitochondria and energy metabolism were detected by Western blotting. ##*p* < 0.01, compared with control group; **p < 0.01, compared with Aβ_25–35_ group, *n* = 3.

Decreased mitochondrial membrane potential (MMP) is a hallmark event in the early stage of apoptosis (54). The results of JC-1 staining showed that the MMP in the Aβ_25–35_ group (*p* < 0.01), while the MMP in the Aβ_25–35_/EGCG, Aβ_25–35_/L-theanine, and Aβ_25–35_/EGCG + L-theanine groups all increased (*p* < 0.01), in which EGCG and L-theanine exhibited a significant synergistic effect (Figures 5A,B). Cellular LDH content increased and ATP production decreased in Aβ_25–35_ group (*p* < 0.01); cellular LDH content decreased in Aβ_25–35_/EGCG + L-theanine and Aβ_25–35_/L-theanine groups (*p* < 0.01) (Figure 5C); compared with Aβ_25–35_ group, cellular ATP production was increased about 4-fold and 6-fold in the Aβ_25–35_/EGCG and Aβ_25–35_/EGCG + L-theanine groups, respectively (Figure 5D). The results indicate that EGCG and L-theanine have different protective effects on mitochondria and have synergistic effects.

The results of mitochondria and energy metabolism-related protein expression detection showed that under Aβ_25–35_ stress conditions, the expression of anti-aging factor Klotho decreased (*p* < 0.01); EGCG and L-theanine alone had no effect, while EGCG + L-theanine up-regulated Klotho expression by about 65% (*p* < 0.01). During calorie restriction, Sirt1 and p-AMPK proteins are highly expressed, while p-mTOR is lowly expressed, closely related to cellular energy metabolism, maintaining the balance of cellular energy supply and demand by affecting multiple links of cellular material metabolism (55, 56). Under Aβ_25–35_ stress conditions, the expression levels of Sirt1 and p-AMPK were decreased, and p-mTOR was significantly increased (*p* < 0.01); EGCG + L-theanine and L-theanine significantly up-regulated Sirt1 and p-AMPK expression levels (*p* < 0.01); in addition, the protein expression level of p-mTOR in the three tea component treatment groups was at a low level (*p* < 0.01) (Figure 5E). The results suggest that L-theanine is more effective than EGCG in activating calorie restriction-like energy metabolism pathways, which maybe related to the high bioavailability of L-theanine (57).

### Transcriptome analysis of the synergistic effect of epigallocatechin gallate and L-theanine in protecting nerve cells

This study obtained 699.87 million raw reads from 15 transcriptome cDNA libraries, and the filtered clean reads accounted for more than 91.23%. Comparing valid readings with Rattus_norvegicu (GCF_000001895.5_Rnor_6.0), each sample had a total contrast of over 81.7% and a unique contrast of over 76.65%. Transcriptome sequencing (RNA-seq) is high quality and suitable for subsequent bioinformatic analysis.

#### Differentially expressed genes enrichment analysis

**FIGURE 6:**
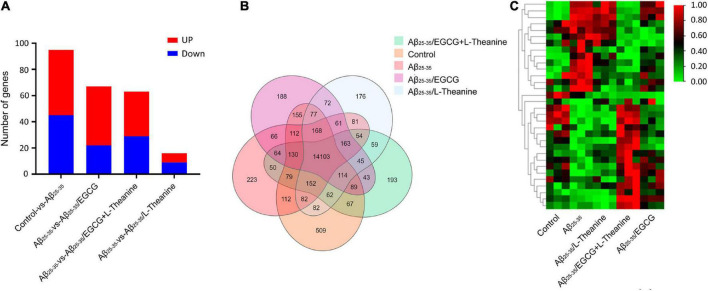
Analysis of differentially expressed genes (DEGs) in different treatment groups. **(A)** Statistical analysis of DEGs expression between groups. **(B)** Venn diagram. One of the circles represents the set of DEGs for a treatment group, areas where different circles were superimposed represent DEGs common to different treatment groups, and non-overlapped parts represent DEGs specific to the treatment group. **(C)** Heat map. The color blocks from red to blue indicate the gene expression levels from high to low.

Differential genes (DEGs) were screened according to the screening conditions of | fold change| ≥ 2 and Q ≤ 0.05. Compared with the control group, 95 DEGs were identified in the Aβ_25–35_ group. Compared with the Aβ_25–35_ group, 67, 63, and 16 DEGs were in Aβ_25–35_/EGCG group, Aβ_25–35_/EGCG + L-theanine group, and Aβ_25–35_/L-theanine group, respectively (Figure 6A). The results of the Venn diagram showed that there were 14103 co-expressed genes in all treatment groups (Figure 6B). Cluster analysis of DEGs of co-expressed genes showed that the expression trends of DEGs in different treatment groups were significantly different, and Aβ_25–35_/EGCG + L-theanine group showed the opposite expression trend to Aβ_25–35_ (Figure 6C).

**FIGURE 7:**
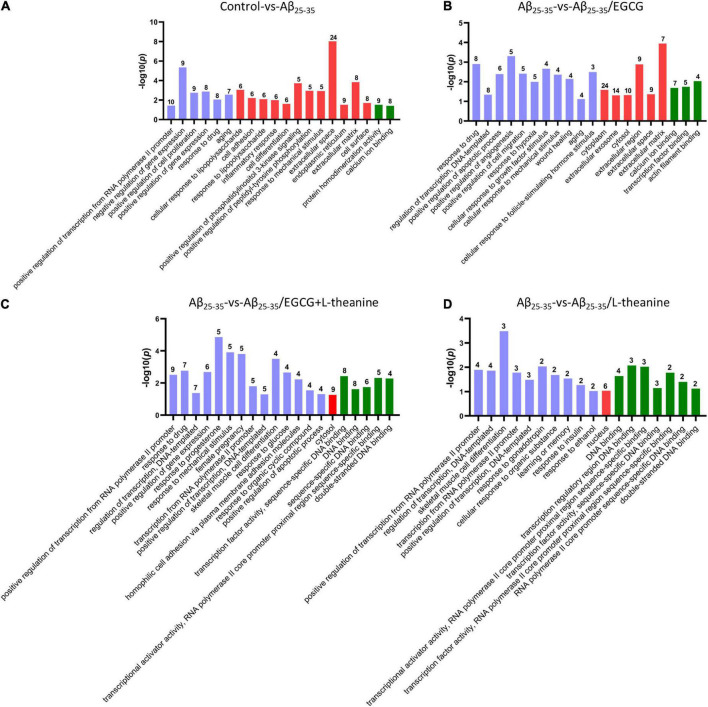
GO enrichment analysis of differentiated genes (DEGs) in PC12 cells of different treatment groups. Graphs from panels **(A–D)** represent the GO analysis of Control-vs-Aβ_25–35_, Aβ_25–35_-vs-Aβ_25–35_/EGCG, Aβ_25–35_-vs-Aβ_25–35_/EGCG + L-theanine, and Aβ_25–35_-vs-Aβ_25–35_/L-theanine, respectively. The blue-purple, red, and green columns in the GO enrichment analysis diagrams represent the biological processes, cellular components, and molecular functions of cells in different treatment groups, respectively.

GO analysis showed that the different genes (DEGs) in Aβ_25–35_ group cells were significantly enriched in transcription, cell proliferation, inflammation, aging, and extracellular matrix (Figure 7A). DEGs in the Aβ_25–35_/EGCG group were mainly enriched in cell translation, extracellular matrix, wound healing, cytoplasm, and growth factor stimulation (Figure 7B). The DEGs-enriched pathways in the Aβ_25–35_/L-theanine group and Aβ_25–35_/EGCG + L-theanine group were similar, mainly enriched in DNA or RNA binding transcriptional activity, gonadotropins, female progesterone and cell differentiation (Figures 7C,D). Progesterone and other estrogenic pathways are neuroprotective against traumatic brain injury by promoting GAP43 expression (58). The above results indicate that Aβ_25–35_ has extensive toxic effects inside and outside cells, while EGCG has extensive intracellular and extracellular repair effects, and L-theanine and EGCG + L-theanine can nourish nerve cells and promote axonal growth, and so on.

#### Interaction network analysis of differential genes and detection of lipid metabolism

**FIGURE 8:**
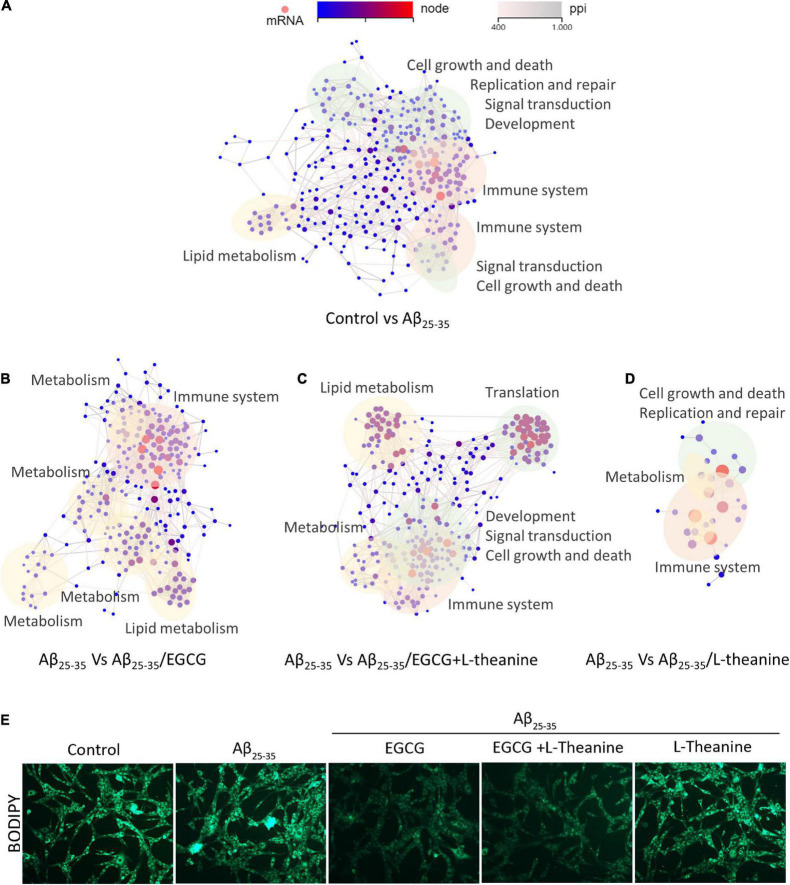
Network interaction plot (PPI) analysis of differential genes in different treatment groups. **(A–D)** Interaction analysis of differential genes in different treatment groups. The yellow blocks in the panel represent metabolisms such as lipids, carbohydrates, amino acids, and energy; green blocks represent signal transduction such as cell growth and death, cellular community and endocrine system, genetic information procession, replication, and repair, etc.; pink blocks represent signal transduction, such as inflammation and immune system. The gray lines represent functional associations of differential genes; the nodes from blue to red represent the ranges of enriched differential genes, from panels **(A–D)** are 1∼64, 1∼58, 1∼45, and 1∼9, respectively. **(E)** BODIPY staining of cell lipid droplets in different treatment groups.

The DEGs interaction network analysis showed that, compared with the control group, the DEGs in the Aβ_25–35_ group (Control-vs-Aβ_25–35_) acted on the following network: metabolism (lipids, carbohydrates, amino acids, and energy metabolism) (Figure 8A yellow blocks), genetic material related (cell growth and death, cellular community and endocrine system, genetic information procession, replication and repair, immune system, and other signal transduction) (Figure 8A green blocks) and the inflammatory immune system (Figure 8A pink blocks). The DEGs of Aβ_25–35_-vs-Aβ_25–35_/EGCG mainly acted on networks such as anti-inflammatory (Figure 8B pink blocks), lipid and energy metabolism pathways (Figure 8B yellow blocks). The DEGs of Aβ_25–35_-vs-Aβ_25–35_/L-theanine mainly act on cell cycle, immune regulation, genetic material such as DNA and RNA (Figure 8C green blocks) and immunomodulation (Figure 8C, pink blocks). The DEGs in Aβ_25–35_-vs-Aβ_25–35_/EGCG + L-theanine mainly interacted in cell cycle, MAPK, metabolism, signal transduction, genetic information processing, and other pathways (Figure 8D yellow, green, and pink blocks). The results indicate that L-theanine fully exerts its regulatory effects on cell cycle, immunity, DNA and RNA, and other genetic materials under EGCG inhibiting Aβ_25–35_ stress and regulating metabolism.

Lipid metabolism is of particular interest due to its high concentration in CNS. Chronic accumulation of phase-separated molecular aggregates such as lipid droplets and amyloid can lead to neurotoxicity (59, 60). The result of lipid droplets BODIPY fluorescence staining showed that under Aβ_25–35_ stress condition, the structure of intracellular lipid droplets became blurred and smaller, or merged into large-area lipid plaques; EGCG and EGCG + L-theanine have significantly reduced the content of intracellular lipid droplets (Figure 8E). This result was corroborated with transcriptomic EGCG promoting lipid and energy metabolism (Figure 8B).

#### Analysis of the synergistic mechanism of epigallocatechin gallate and L-theanine from the transcriptome level

**FIGURE 9:**
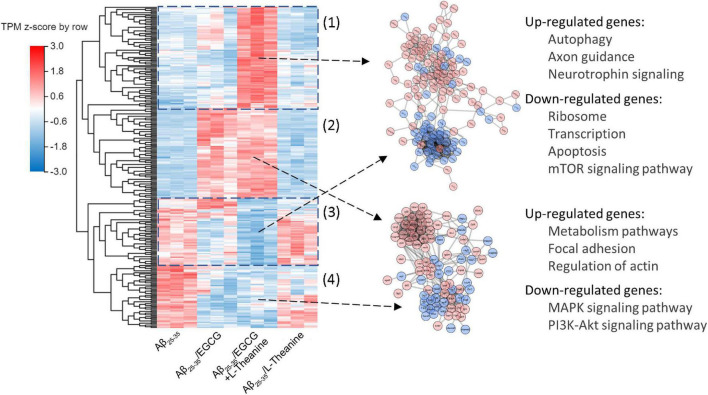
Analysis of the synergistic mechanism of EGCG and L-theanine from the transcriptome level. (1–4) The results showed that the cluster map of DEGs in the Aβ25-35-vs-Aβ25-35/EGCG + L-theanine was mainly divided into four blocks, of which (1) and (3) blocks gene expression trend was opposite to that of other treatment groups, and (2) and (4) blocks gene expression trend were very similar to that of Aβ_25–35_-vs-Aβ_25–35_/EGCG. The expression level of each gene was calculated using the transcripts per kilobase of exon model per million mapped reads (TPM) after normalizing the expression level of each gene.

Using the DEGs of Aβ_25–35_-vs-Aβ_25–35_/EGCG + L-theanine as a reference, the cluster analysis and interaction correlation of different genes between groups were carried out. The results showed that the cluster map of DEGs in the Aβ_25–35_-vs-Aβ_25–35_/EGCG + L-theanine was mainly divided into four blocks, of which two blocks [Figure 9 (1,3)] gene expression trend was opposite to that of other treatment groups, which was presumed to result from the synergistic interaction between EGCG and L-theanine. The up-regulated genes were mainly related to autophagy, axon guidance and neurotrophin signaling pathways, while the down-regulated genes were mainly enriched in ribosome, transcription, apoptosis and mTOR signaling pathway. This result is consistent with the previous experimental results.

The gene expression trends of the other two blocks [Figure 9 (2,4)] were very similar to that of Aβ_25–35_-vs-Aβ_25–35_/EGCG. It is speculated that these two blocks were mainly the effects of EGCG, and the up-regulatory genes were mainly enriched in metabolism pathways, focal adhesion and actin regulation, and the down-regulated genes were main enriched in MAPK and PI3K-Akt signaling pathways. Combined with the previous results (Figure 8), we speculated that under the premise that EGCG inhibits amyloid stress, promotes lipid metabolism and inhibits cell mitogenic activity, L-theanine could exert anti-inflammatory, promote axonal growth and nourish nerves. The synergistic effect of EGCG and L-theanine made EGCG have a milder effect on cells, thereby better maintaining the high-fidelity structure of cellular proteins (Figure 3C).

## Discussion

This study demonstrates that EGCG and L-theanine synergistically promote repair and regeneration of differentiated neural cell line PC12 cells under amyloid stress conditions. EGCG mainly inhibits amyloid stress and inflammation and promotes lipid metabolism. In the presence of EGCG, L-theanine can fully exert its neurotrophic effect. EGCG + L-theanine targets the cell cycle, keeps nerve cells in a quiescent state under amyloid stress, promotes cell viability and axonal growth, and maintains the high-fidelity structure of cellular proteins.

### Maintain the fidelity structure of nerve cell proteins

The toxicity of disease-causing protein aggregates may be due to their inherent misfolded nature and structural heterogeneity. These properties will lead to numerous aberrant interactions with various cellular components, including phospholipid bilayers, protein receptors, soluble proteins, RNA, and small metabolites, leading to cellular damage and ultimately cell death (38). Extensive literature studies have shown that EGCG binds to fold-rich aggregates, significantly inhibits β-amyloid fibrillogenesis, and is a potent remodeler of mature amyloid fibrils (16, 17). EGCG targeted and inhibited β-sheets formation of Aβ_25–35_ toxic structure (Figure 2C), and promoted the growth of differentiated PC12 cells (Figure 2A). Aβ_25–35_ can function as a neurotrophic factor for differentiation neurons and has important physiological roles (61, 62). Aβ_25–35_ may have a certain neurotrophic effect after EGCG changes its toxic structure, which needs to be further verified.

Under Aβ_25–35_ stress conditions, EGCG, L-theanine, and EGCG + L-theanine significantly downregulated neurotoxic proteins such as p62, ubiquitination and 4-HNE, and inhibited inflammatory pathways (Figures 3A,B). L-theanine significantly suppressed the shift of the protein infrared spectrum to lower wavenumbers, while also protecting the disordered structure of the protein (Figure 3C). Hub proteins, such as GAP43 and ubiquitination, are often disordered and flexible structures that perform essential signal transduction (63). In many neurodegenerative diseases, intrinsically disordered proteins form fibrillar aggregates for pathogenesis (64). EGCG + L-theanine maintained the high-fidelity structure of the neuronal protein, and the disordered structure was the same as that of the control group (Figure 3C). EGCG has a significant inhibitory effect on amyloid stress and significantly affects the protein amide I band spectrum, indicating that EGCG also has a more significant effect on cells. The Aβ_25–35_/EGCG + L-theanine group has the best cellular protein fidelity, almost identical to the control group (Figure 3C). This result may be that L-theanine’s weak acidity helps protect EGCG from being degraded, making EGCG bind more tightly to Aβ_25–35_, and enhancing its anti-amyloid stress activity (65).

Based on the above results, we speculate that EGCG and L-theanine can promote each other, and fully exert repair and growth-promoting effects, so that EGCG + L-theanine exhibits milder and more effective cytoprotective activities.

### Targets cell cycle and promotes the formation of cellular quiescence

Cell mitotic quiescence is critical for complex connected neuronal systems (66, 67). In differentiated neuronal cells, amyloid stress leads to abnormal cell cycle entry and is closely associated with neuronal inflammation and apoptosis (68–70). This study showed that the expression of cyclin marker proteins Cyclin D1 and Cyclin B1 in Aβ_25–35_ group was significantly increased. In Aβ_25–35_/EGCG + L-theanine group, the expression of Cyclin D1 and Cyclin B1 was significantly decreased, the percentage of cell cycle G0/G1 phase was significantly increased, the cell inflammation was down-regulated, the GAP43 protein expression was up-regulated, the p-mTOR expression was down-regulated, and the axonal growth was obvious (Figures 3, 4). In quiescent cells, mTOR is deactivated, and levels of pS6, cyclin D1, p21, and p16 are low (52). This study and literature show that EGCG + L-theanine promotes the formation of the neuronal quiescent state under amyloid stress, keeps differentiated neuronal cells in the G0 phase, and improves cell viability and axonal growth.

Aβ_25–35_ significantly promoted the formation of neuronal inflammation and oxidative stress, while EGCG and EGCG + L-theanine had significant inhibitory activity (Figures 3A,B). The transcriptome results further demonstrated that EGCG + L-theanine enhanced the anti-inflammatory efficacy (Figures 8, 9). Additionally, lipids play an important role in neurodegeneration, neuroinflammation, and psychiatric disorders. Imbalances in sphingolipid levels is associated with diseases (71). EGCG and EGCG + L-theanine significantly promote cellular lipid metabolism, energy metabolism, and caloric restriction (Figures 5E, 8E). These results further demonstrate that EGCG and EGCG + L-theanine promote a quiescent state of neuronal cells under amyloid stress conditions.

### Synergistic effects of epigallocatechin gallate and L-theanine in promoting nerve cell regeneration

Epigallocatechin gallate exhibited toxicity at high concentrations, while L-theanine exhibited cell growth-promoting activity. Under Aβ_25–35_ stress conditions, EGCG promoted cell viability concentration-dependent, while L-theanine showed no cytoprotective activity. The bioavailability of EGCG is not high, but it has a significant anti-amyloid stress effect. L-theanine is a naturally brain-derived neurotrophic factor (BDNF) but has no inhibitory activity against amyloid stress (Figures 2A,B). The above results indicate that the mechanisms of action of EGCG and L-theanine have opposite effects.

Neurotrophic factors play an important role in combinatorial approaches to spinal cord repair by promoting cell survival, and maintaining axonal growth and regeneration (72). Although neurotrophins have positive effects in animal models, their short half-lives, low bioavailability, and limited permeability across the blood-brain barrier (BBB) limit their use in patients (73). The bioavailability of L-theanine is nearly 100%, and it has BDNF and neurogenesis (23, 29, 74–76). The transcriptome results showed that L-theanine mainly acts on genetic material such as DNA and RNA, and has estrogen-like and other growth-promoting effects (Figures 7–9). Their estrogenic pathways have significant neuroprotective effects.

Neuronal cells have abundant mitochondria, especially in axonal structures. Differentiated neural cells have higher energy requirements than normal cells, which require the transport of mitochondria to distal synapses (77). Therefore, protecting mitochondria, improving energy metabolism, and maintaining the differentiated state of neuronal axons are crucial. EGCG promoted the metabolism of lipids, carbohydrates, amino acids and energy (Figures 8B,E), and significantly promoted ATP production (Figure 5D). L-theanine inhibited LDH production and significantly promoted the phosphorylation of AMPK. EGCG + L-theanine significantly up-regulated Klotho protein expression, had the best effect on improving mitochondrial membrane potential activity and promoted mitochondrial rejuvenation (Figure 5).

**FIGURE 10:**
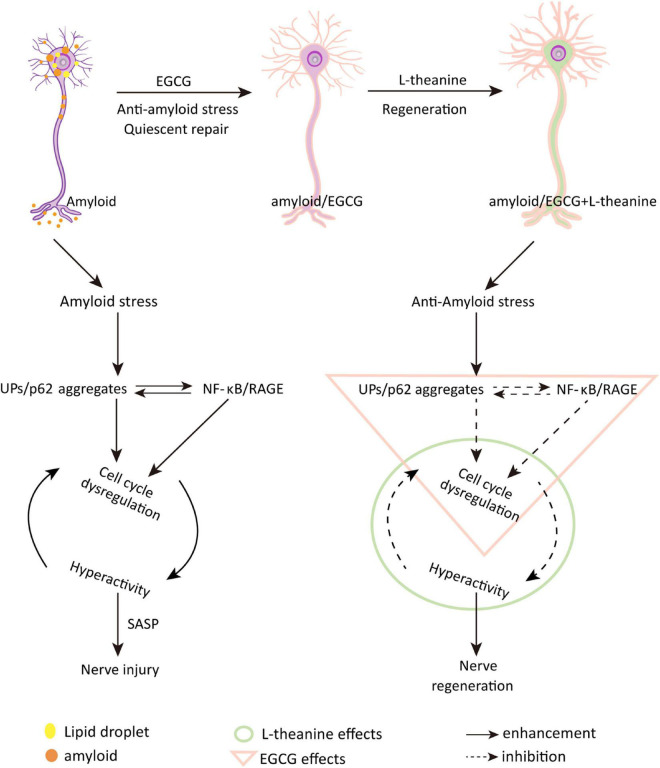
Schematic diagram of the molecular mechanism by which EGCG and L-theanine synergistically promote nerve cell repair and regeneration under amyloid stress conditions. Epigallocatechin gallate (EGCG) and *N*-ethyl-L-glutamine (L-theanine) are the representative functional components of green tea (Camellia sinensis). This study investigated the synergistic protective effect of EGCG and L-theanine on neural cells by establishing an AD model of Aβ_25–35_-induced differentiated neural cell line PC12 cells. Through a series of cell and molecular biology experiments, it was found that amyloid stress mainly promotes the accumulation of UPs/p62-modified aggregates, upregulates the NF-κB/RAGE inflammatory pathway, stimulates cell-cycle dysregulation, and leads to cell hyperactivity and damage; EGCG + L-theanine can repair and regenerate amyloid-stressed differentiated neural cells. The synergistic mechanism is that under the premise that EGCG inhibits amyloid stress and inflammation and promotes metabolism, L-theanine can nourish nerves. EGCG + L-theanine increases the G0/G1 phase, down-regulates the expression of cyclins, keeps the cells in a quiescent state, and is beneficial to the repair and regeneration of the differentiated neuronal cells. In addition, EGCG + L-theanine maintains the high-fidelity structure of cellular proteins. This study revealed for the first time that the synergistic effect of EGCG with L-theanine or drinking green tea may be an effective way to promote nerve cell repair and regeneration and slow the progression of AD.

The above results revealed that under the premise that EGCG binds to amyloid and promotes quiescent state repair of cells, L-theanine enters cells to play neuron nutrition and promote axonal growth (Figure 10). Drink 5–10 g of green tea daily to get adequate amounts of EGCG and L-theanine (15, 22). An epidemiological survey study showed that drinking two or more cups of green tea per day (100 mL/cup) can reduce the damage to brain memory (78). Another survey found that drinking two cups of tea daily or drinking tea continuously for more than 8–10 years can reduce the risk of Parkinson’s disease by 28˜80% (79).

## Conclusion

Proteotoxic stress, mitochondrial dysfunction, and genomic instability lead to cellular hyperactivity and are closely associated with age-related degenerative diseases such as atherosclerosis, type 2 diabetes, osteoporosis, and Alzheimer’s disease. In the context of neurodegenerative diseases, targeting a single pathway may not be sufficient to promote long-distance axonal regeneration and functional recovery after injury. Amyloid stress-mediated neuronal degeneration results from multi-faceted effects and the synergy effect of EGCG and L-theanine exhibits multi cytoprotective activities. EGCG mainly inhibits the formation of amyloid protein rich in toxic β-sheet structure, improves lipid and energy metabolism, protects cell membrane structure, and maintains cellular protein homeostasis (Figures 3–5, 8). L-theanine mainly improves the endogenous antioxidant capacity of cells and protects mitochondria and cell genetic materials (Figures 5, 8). The synergistic effect of EGCG and L-theanine has multi-target protective effects on nerve cells under amyloid stress. EGCG and L-theanine promote each other and exert mild cytoprotective effects. This study revealed one of the core underlying mechanisms by which green tea delays age-related degenerative diseases such as AD.

## Data availability statement

The original contributions presented in the study are publicly available. This data can be found here: NCBI BioSample database, accession SAMN29216840.

## Author contributions

SC performed writing the original draft. ZL and SC performed funding acquisition. XX, JW, XZ, MF, JY, WP, and BH performed methodology. ZL performed supervision. All authors have read and agreed to the published version of the manuscript.

## Footnotes

1
www.genomics.cn

2
https://cn.string-db.org/

## Abbreviations

Aβ_25–35_: β -amyloid fragment 25-35
EGCG: (-)-epigallocatechinegallate
L-theanine: *N*-ethyl -L-glutamine
FTIR: Fourier transform infrared spectroscopy
ThT: thioflavin T
TEM: transmission electron microscope
MTT: 3-(4,5-dimethyl-2-thiazolyl)-2,5-diphenyl tetrazolium bromide
ATP: adenosine triphosphate
ROS: reactive oxygen species
GAP43: growth associated protein-43
TNF-α: tumor necrosis factor-α
BACE1: beta-site amyloid cleaving enzyme-1
Aβ42: amyloid 1-42
IL-6: interleukin-6
NF-kB: nuclear factor kappa-B
mTOR: mammalian target of rapamycin
P-mTOR: phospho-mammalian target of rapamycin
Sirt1: Sirtuin1
UPs: ubiquitin conjugated proteins
4-HNE: 4-hydroxynonenal
RAGE: receptor for advanced glycation endproducts
p62: sequestosome 1
SASP: the senescence-associated secretory phenotype
MMP: mitochondrial membrane potential
LDH: lactate dehydrogenase
JC-1: it is an ideal fluorescent probe widely used to detect mitochondrial membrane potential
DAPI: It is a fluorescent dye that can bind strongly to DNA and is often used in fluorescence microscopy
TPM: trans per kilobase of exon model per million mapped reads
BDNF: brain-derived neurotrophic factor
50 μM Aβ_25–35_: Aβ_25–35_
50 μM Aβ_25–35_/50 μM EGCG: Aβ_25–35_/EGCG
50 μM Aβ_25–35_/50 μM L-theanine: Aβ_25–35_/L-theanine
50 μM Aβ_25–35_/50 μM EGCG + 50 μM L-theanine: Aβ_25–35_/EGCG + L-theanine.
